# Consequences of Vibrational Strong Coupling on Supramolecular Polymerization of Porphyrins

**DOI:** 10.1021/jacs.4c02267

**Published:** 2024-04-20

**Authors:** Kripa Joseph, Bas de Waal, Stef A. H. Jansen, Joost J. B. van der Tol, Ghislaine Vantomme, E. W. Meijer

**Affiliations:** † Institute for Complex Molecular Systems, Laboratory of Macromolecular and Organic Chemistry, Eindhoven University of Technology, PO Box 513, 5600 MB, Eindhoven, The Netherlands

## Abstract

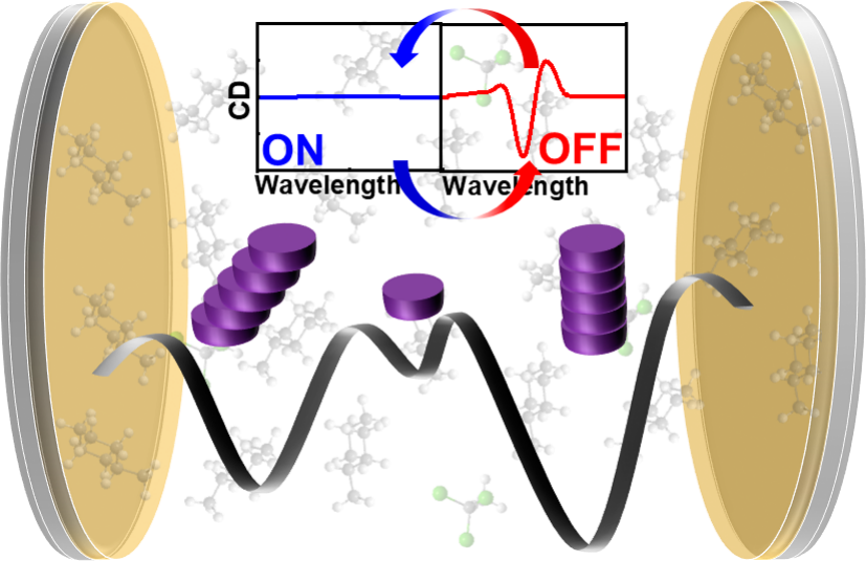

Supramolecular polymers display interesting optoelectronic properties and, thus, deploy multiple applications based on their molecular arrangement. However, controlling supramolecular interactions to achieve a desirable molecular organization is not straightforward. Over the past decade, light–matter strong coupling has emerged as a new tool for modifying chemical and material properties. This novel approach has also been shown to alter the morphology of supramolecular organization by coupling the vibrational bands of solute and solvent to the optical modes of a Fabry–Perot cavity (vibrational strong coupling, VSC). Here, we study the effect of VSC on the supramolecular polymerization of chiral zinc-porphyrins (***S-*Zn**) via a cooperative effect. Electronic circular dichroism (ECD) measurements indicate that the elongation temperature (*T*_e_) of the supramolecular polymerization is lowered by ∼10 °C under VSC. We have also generalized this effect by exploring other supramolecular systems under strong coupling conditions. The results indicate that the solute–solvent interactions are modified under VSC, which destabilizes the nuclei of the supramolecular polymer at higher temperatures. These findings demonstrate that the VSC can indeed be used as a tool to control the energy landscape of supramolecular polymerization. Furthermore, we use this unique approach to switch between the states formed under ON- and OFF-resonance conditions, achieved by simply tuning the optical cavity in and out of resonance.

## Introduction

Supramolecular polymerization is a ubiquitous phenomenon in nature.^1^ The adaptive and dynamic nature of supramolecular polymers has opened a new portal to optimize functional materials.^2^ Noncovalent interactions (solute–solute, solute–solvent, and solvent–solvent) holding the monomeric units together are responsible for the dynamic nature of supramolecular polymers, which enables them to be highly sensitive to external stimuli and other factors, such as solvent composition,^3,4^ temperature,^5,6^ light,^7,8^ and pH.^9,10^ The thermodynamic stability of a supramolecular polymer is determined not only by the properties of the solute but also by the cohesive and dispersion forces of the solvent.^11^ Hence, the enthalpic and entropic contributions to the free energy of a supramolecular polymer strongly depend on the polarity of the solvent.^3^ However, pure solvents offer a limited scope for tailoring the properties of aggregates, although a combination of solvents is an alternative. Here, we employ a novel concept of light–matter strong coupling to control solute–solute, solute–solvent, and solvent–solvent interactions and thereby modify supramolecular polymerization. We also exploited this new concept of light–matter strong coupling to switch between the different states of supramolecular polymerization without any chemical or real photon as input but by merely controlling the vacuum fluctuations.

**Figure 1:**
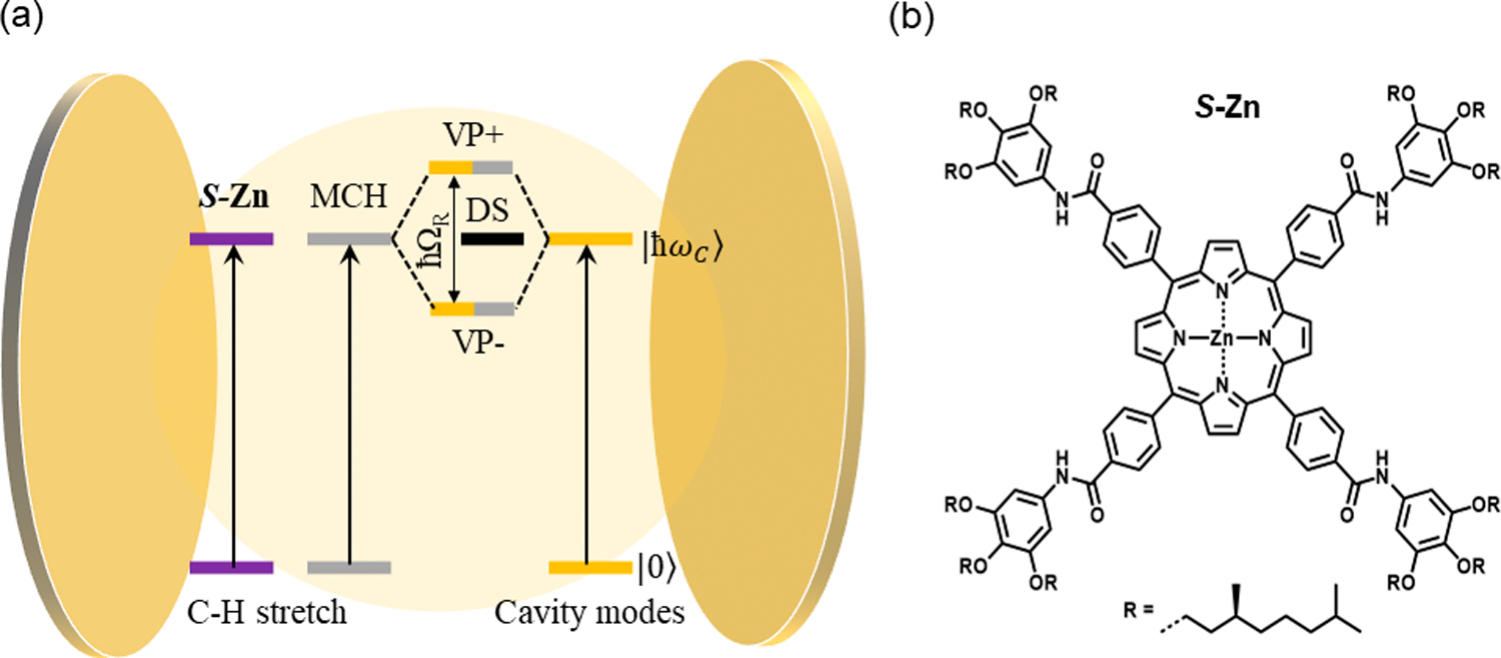
(a) Schematic illustration of vibro-polaritonic states *VP*+, *VP*–, and *N*-1 DS formed due to vibrational strong coupling (via cooperative effect) between the molecular vibrational bands and the resonant optical mode of a Fabry–Perot cavity. (b) Molecular structure of the monomer ***S*-Zn**.

Over the past years, light–matter strong coupling has generated considerable interest in modifying molecular and material properties such as chemical reactivity,^12−14^ transport,^15,16^ and supramolecular assembly.^17−20^ An example of the ubiquity of light–matter coupling in nature is the recent report on the existence of self-hybridized polaritonic states in water droplets via ultrastrong coupling.^21^ For any system to be in the strong coupling regime, the molecular and optical modes at resonance must exchange virtual photons faster than the dissipative processes. This leads to the formation of hybrid light–matter states, also called polaritonic states. Such a coupling is possible even in the dark due to the interaction with zero-point energy fluctuations. Coupling the electronic transitions (electronic strong coupling, ESC) or vibrational bands (vibrational strong coupling, VSC) has been shown to alter the molecular and chemical properties.^22^ Under the VSC, vibro-polaritonic states are formed (Figure 1a). Since the *N* number of molecules are coupled to an optical mode, *N*+1 hybrid states are formed. These include the bright states, or the upper and lower polaritonic states (*VP*+ and *VP*−), which are separated by an energy called Rabi-splitting energy (ℏΩ_*R*_). In addition to the bright states, *N*–1 dark states (DS) are also formed. Experimentally, a system is said to be strongly coupled when the Rabi-splitting energy is larger than the full-width half-maximum (fwhm) of both the optical mode and the molecular transition. VSC has been shown to modify chemical energy landscapes and thus chemical reactivity.^23^ It is known that symmetry has a role in determining its effect on chemical systems.^24,25^ Nevertheless, many aspects of VSC are still unclear; hence, detailed experiments are required to get a better insight into the effect.

As supramolecular systems are highly sensitive to their environment, a systematic study of them under VSC will provide better insight into the fundamentals of polaritonic chemistry. Recently, it was shown that VSC can modify the morphology of supramolecular assemblies^17,18^ and the pseudopolymorphism of metal–organic frameworks.^19^ These reports, together with the recent literature,^26^ emphasize that the noncovalent interactions such as hydrogen bonding together with π–π stacking are modified by VSC. The effect of VSC on solute–solute, solute–solvent, and solvent–solvent interactions can be further tailored in supramolecular systems to favor certain pathways without any chemical input. In this work, we study the effect of VSC on the supramolecular polymerization of chiral zinc-porphyrins (***S-*Zn**, Figure 1b) in methylcyclohexane (MCH). Since the monomer concentration is low in the supramolecular system studied, it is difficult to reach the strong coupling regime by directly coupling the solute to the optical mode. Therefore, we apply the concept of cooperative coupling,^13,17,18,26,27^ where the solute can be strongly coupled to the optical mode via the solvent, if the solute and solvent have overlapping vibrational bands, as illustrated in Figure 1a. Through electronic circular dichroism (ECD) measurements, we demonstrate that the elongation temperature (*T*_e_) is lowered under VSC, indicating that strong coupling destabilizes the supramolecular polymer at higher temperatures. To generalize the study, we also explored the effect of VSC on different supramolecular systems: *S*-3,7-dimethyloctylamine-triphenylamine trisamide (***S-*TPA**) and *S*-triazine-1,3,5-tribenzenecarboxamide (***S-*T**_**N**_). It is evident from the results that the VSC can tune the solute–solvent interactions and thus favor different states of supramolecular polymerization at a particular temperature. Also, the results highlight the importance of cooperative effect in such studies, as reported previously.^13,17−19^ Finally, we demonstrate that switching between different states of the supramolecular energy landscape is possible by carefully tuning the optical cavity in and out of resonance.

## Results and Discussion

### Supramolecular Polymerization of *S-*Zn

The monomer, ***S-*Zn**, is well-studied for supramolecular polymerization and is known to show pathway complexity.^4,28,29^ Previous reports have demonstrated that ***S-*Zn** in MCH assembles into long cofacial chiral H-aggregates via a cooperative mechanism which can be confirmed by the hypsochromic shift of the absorption band and the appearance of a strong bisignate Cotton effect at 393 nm upon cooling (Figure S1).^28^ The formation of 1D supramolecular H-aggregates is driven by hydrogen-bonding and van der Waals interaction as well as π–π stacking. To a lesser extent, ***S-*Zn** also polymerizes into J-aggregates via an isodesmic mechanism, which can be followed by the formation of a broad absorption band around 425 nm.

### Vibrational Strong Coupling of *S-*Zn via Cooperative Coupling

**Figure 2:**
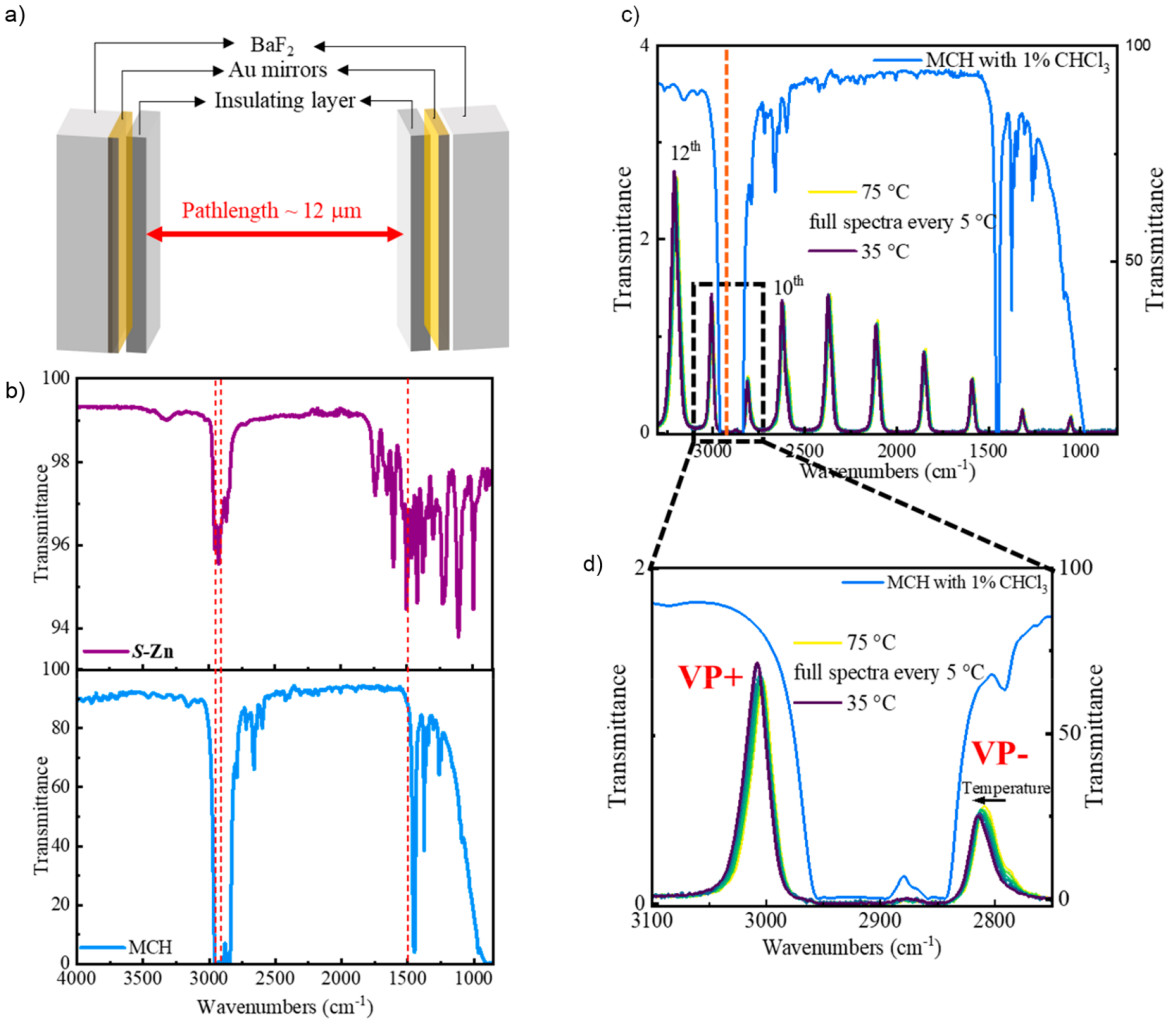
(a) Schematic illustration of Fabry–Perot cavity used for the experiments. (b) FT-IR transmission spectra of *S***-Zn** (top) and MCH (bottom), red dashed lines show the overlapping bands between the solute and solvent. (c) FT-IR transmission spectra of ON-resonance cavity measured in intervals of 5 °C from 75 to 35 °C (blue trace corresponds to the FT-IR spectrum of MCH, and the orange dashed line indicates the calculated frequency of 11th optical mode that has been coupled to the vibrational bands of MCH, giving rise to the vibro-polaritonic states). The insignificant shift in the frequency of optical modes indicates that the effect of temperature on strong coupling condition is negligible. (d) FT-IR transmission spectra showing the vibro-polaritonic states formed when the optical mode is strongly coupled to the vibrational bands of MCH around ∼2900 cm^–1^.

To study the effect of the VSC on the supramolecular polymerization of ***S*****-Zn**, a microfluidic tunable optical Fabry–Perot (FP) cavity was used. The FP cavity consists of two parallel mirrors, which are fabricated by sputtering 10 nm of Au on IR transparent (BaF_2_) substrates (Figure 2a). To avoid the interaction of molecules with Au mirrors, the mirrors are insulated with a 100 nm thick layer of poly(vinyl alcohol) (PVA). The two mirrors are then separated by a 12 μm thick Mylar spacer and assembled into a tunable microfluidic cell (Figure S2). The solution of ***S-*Zn** in MCH is injected into a pretuned FP cavity to reach the strong coupling regime. The FT-IR transmission spectrum of the pretuned empty cavity is shown in Figure S2b,c. For control experiments, we prepared NON-cavities by insulating the BaF_2_ substrates directly (without Au mirrors) with PVA. Note that NON-cavities are analogous to the cuvettes with an optical path length of a few μm. The OFF-resonance cavity, in which the optical modes are tuned away from the vibrational bands, serves as an additional control experiment. The reference measurements take into consideration the artifacts due to physical confinement or interaction with the Au film. The role of PVA on supramolecular polymerization of ***S-*Zn** as a potential hydrogen-bond scavenger^30^ is negligible in our experiments, as is clear from the experiments carried out with SiOx as insulation film (Figure S3). In an ON-resonance cavity, the spacing between the mirrors is finely tuned such that the optical mode is in resonance with the vibrational band at normal incidence,^22,31^ leading to the formation of vibro-polaritonic states.

FT-IR Transmission spectra of ***S-*Zn** and MCH are shown in Figure 2b. Note that the (CH_2_)_4_ symmetric and antisymmetric stretching frequencies and CH_3_ antisymmetric stretches of MCH are around 2850, 2920, and 2950 cm^–1^,^32^ respectively, as shown in Figure 2b (blue trace). Due to the low concentration of ***S-*Zn**, it is not possible to achieve strong coupling conditions by directly coupling ***S-*Zn** to the optical modes; hence, we apply the concept of cooperative effect (Figure 1a). As can be seen in Figure 2b, C–H stretching frequencies of ***S-*Zn** and MCH close to 2920 cm^–1^ are overlapping and, hence, are viable for cooperative coupling. The ON-resonance condition is achieved by coupling the 11th optical mode of the FP cavity to the C–H stretching frequencies of ***S-*Zn** and MCH via cooperative effect (Figure 2c, vibro-polaritonic states are zoomed in Figure 2d). *VP*+ and *VP*– are separated by an energy (Rabi splitting, ℏΩ_*R*_) of 193 cm^–1^, which is larger than the fwhm of the vibrational bands of both MCH (100 cm^–1^) and the optical mode (35 cm^–1^). The first result shows that no change is observed in the supramolecular polymerization of ***S*****-Zn** in MCH under VSC, and the *T*_e_ remains the same as that in the cuvette (Figure S1) and ON-resonance cavity (Figure S4).

**Figure 3:**
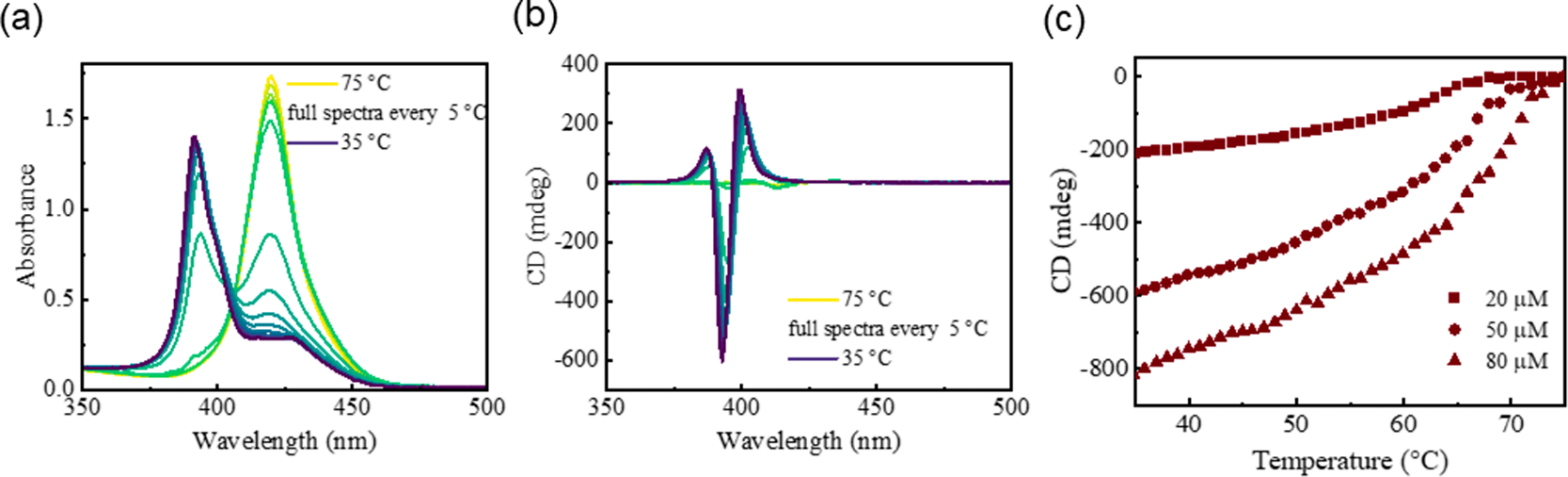
Cuvette measurements. (a) VT-absorption and (b) VT-ECD spectra of *S***-Zn** in MCH with 1% (v/v) CHCl_3_, measured in the interval of 5 °C from 75 to 35 °C with a cooling rate of 1 °C min^–1^. (c) ECD cooling curves of *S***-Zn** in MCH with 1% (v/v) CHCl_3_, at different concentrations, are plotted.

### VSC lowers *T*_e_ of supramolecular polymerization of *S-*Zn

Previously, it was shown that the competing aggregation pathways increase the responsiveness of the supramolecular system to environmental factors.^4^ Indeed, pathway complexity of *S***-Zn** can be controlled by the addition of good solvent (such as chloroform, CHCl_3_), favoring the presence of J-aggregates and changing the kinetic behavior of the cooperatively formed H-aggregates.^4^ On the basis of this report,^4^ we repeated the cavity experiments in a mixture of solvent MCH with 1% (v/v) CHCl_3_. Note that CHCl_3_ also has C–H stretching frequencies (∼3010 cm^–1^, Figure S5)^33^ in close proximity to that of ***S-*Zn** and MCH.

**Figure 4:**
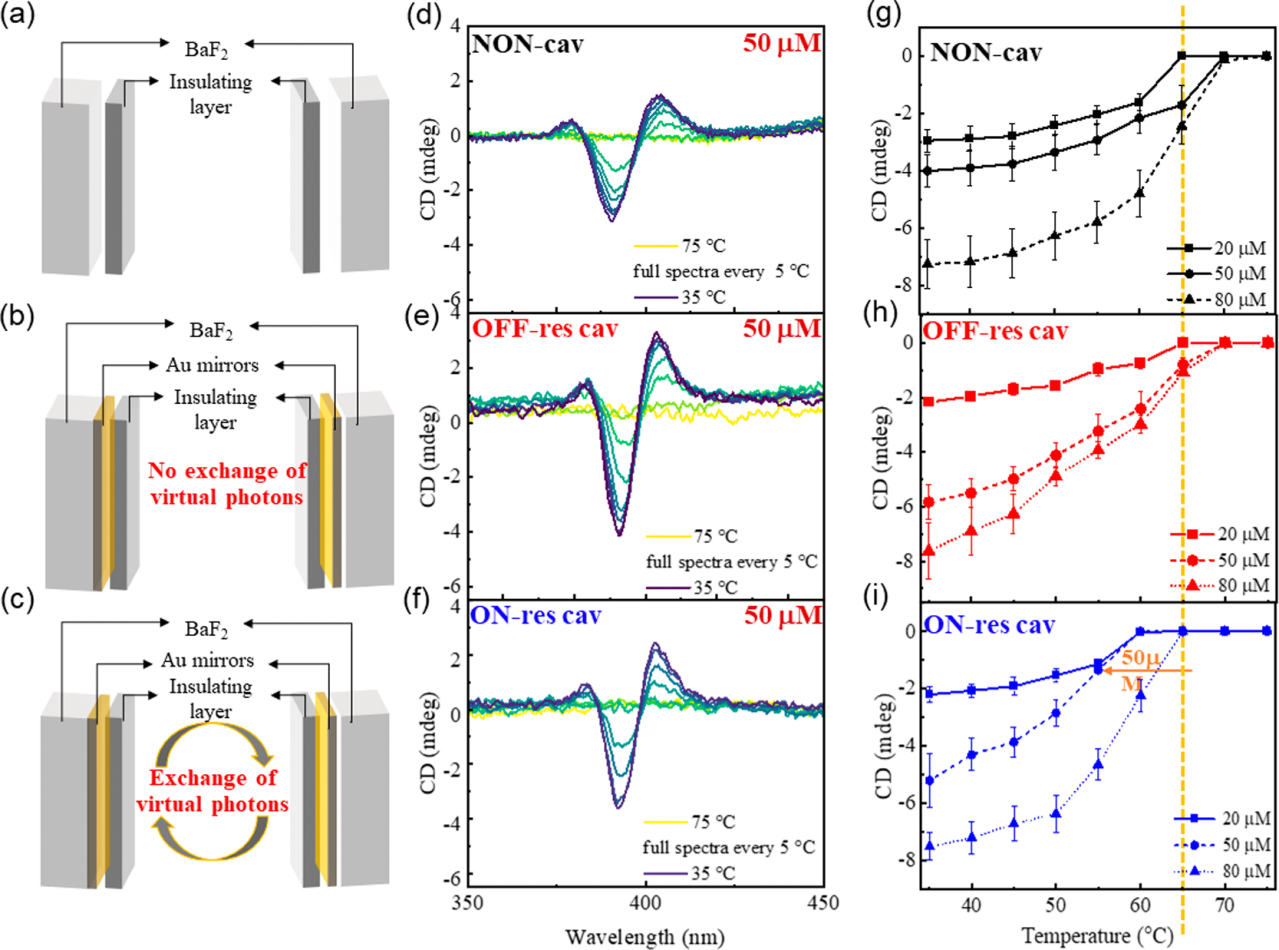
Schematic illustration of (a) NON-, (b) OFF-resonance, and (c) ON-resonance cavities, and the corresponding VT-ECD spectra of ***S-*Zn** (conc = 50 μM) in MCH with 1% (v/v) CHCl_3_, are shown in (d), (e), and (f). Cooling curves of ***S-*Zn** measured in (g) NON, (h) OFF-resonance cavities, and (i) ON-resonance cavities are plotted as a function of concentration. All the spectra are measured in the interval of 5 °C from 75 to 35 °C with a cooling rate of 1.7 °C min^–1^. Yellow dashed line goes through the *T*_e_ of control experiments (50 μM).

At 50 μM concentration of ***S*****-Zn** in MCH (with 1% (v/v) CHCl_3_), the *T*_e_ is determined to be ∼67 °C in a cuvette. Figure 3a–c show the VT-absorption and VT-ECD measurements carried out in a cuvette (optical path length of 1 mm) with a cooling ramp of 1 °C min^–1^. We repeated the VT-ECD measurements in NON, OFF, and ON-resonance cavities, and schematic illustrations of the corresponding cavities are shown in Figure 4a–c. As the BaF_2_ windows are also transparent in the UV–visible region, it is possible to follow the ECD spectra of *S***-Zn** in the spectropolarimeter together with monitoring the strong coupling condition by recording the FT-IR spectra in the mid-IR region. Measurements were performed in NON, OFF, and ON resonance cavities at a cooling rate of 1.7 °C min^–1^ using a Specac temperature controller; Figure 4d–f correspond to their VT-ECD spectra (conc = 50 μM). In NON- and OFF-resonance cavities (Figure 4d,e), we observed that the progression of self-assembly and the elongation temperature (70 °C < *T*_*e*_ < 65 °C) are similar to those observed in cuvette measurements (Figure 3b,c) and literature reports.^4,28^

In the ON-resonance cavity, when the vibrational band remove at around 2900 cm^–1^ is strongly coupled to the optical mode (Figure 2c,d), no change in the ECD spectral profile of the assembly is observed. However, the *T*_e_ of supramolecular polymerization is lowered by around 10 °C (to the range of 60 °C < *T*_e_ < 55 °C), as shown in Figure 4f,i. Note that the effect of temperature on the frequency of optical modes is negligible, as is evident from Figure 2c,d. Recently, Zhong et al. reported that VSC enables coassembling of DNA origami at 2 °C below the required thermal conditions.^20^

To see if VSC has an effect on the mechanism of supramolecular polymerization of ***S-*Zn**, we also repeated the experiments as a function of concentration under different conditions (NON, OFF, and ON-resonance cavities, Figure 4g–i). Due to the limited number of data points, it was difficult to gain insight into the mechanism from fitting of a mass-balance model. However, we observed that the effect of VSC depends on the monomer concentration. For a 20 μM concentration of ***S-*Zn**, compared to the NON- (Figure S6a) and OFF-resonance cavities (Figure S6b), we observed that the *T*_e_ is lowered by only ∼5 °C in the ON-resonance cavity (Figure S6c). While for the 80 μM solution of ***S-*Zn**, compared to NON- and OFF-resonance cavities, *T*_e_ is lowered by at least 10 °C in the ON-resonance cavity. The influence of monomer concentration on the effect of VSC is also clear from the cooling curves shown in Figure 4g–i, which are measured at different concentrations under different conditions. These results indicate that coupling the vibrational bands of MCH, ***S-*Zn** and CHCl_3_ (the latter ones by means of cooperative effect) to the optical mode alters the solvent–solvent, solute–solvent, and solute–solute interactions.^17,18,26^ This might modify the hydrogen bonding, van der Waals interactions, and π–π stacking along the polymer backbone^3^ leading to the destabilization of H-aggregates at higher temperatures.

To further confirm the effect of VSC and understand the role of solute and good solvent (CHCl_3_) in determining the effect of VSC, control experiments of ***S-*Zn** in deuterated methylcyclohexane (MCH-*d*_14_) and a solvent mixture of MCH-*d*_14_ with 1% (v/v) CDCl_3_ were carried out. Cuvette measurements of ***S-*Zn** in MCH-*d*_14_ are shown in Figure S7a, b, and d. The aliphatic C–H stretch of ***S-*Zn** around ∼2900 cm^–1^ does not overlap with the vibrational bands of MCH-*d*_14_ (Figure S7c). Under VSC of the C–D stretch of solvent alone (Figure S7e), no change in *T*_e_ was observed (Figure S8). Figure S9 shows the cuvette measurements from the latter control experiment. As can be seen from Figure S10, no change in the *T*_e_ is observed for the supramolecular polymerization of ***S-*Zn** in MCH-*d*_14_ with 1% (v/v) CDCl_3_. This is also proposed to be due to the absence of overlapping vibrational bands between the solute, the bad solvent, and/or the good solvent. These observations reveal that the destabilization of H-aggregates in nondeuterated solvents at higher temperatures is indeed due to VSC. These experiments also highlight the importance of strongly coupling the solute and the good solvent by cooperative coupling in determining the effect of VSC on the supramolecular polymerization of ***S-*Zn**.

In order to generalize the effect of the VSC on supramolecular polymerization, we further extended this approach to different supramolecular systems. For triazines (***S**-***T**_**N**_, Figure S11a) in tetrachloroethane (TeCE), there is no overlap between the vibrational bands of solute and solvent as can be seen from Figure S11b. Under VSC (Figure S11c and d), the *T*_e_ remains the same as observed in the control experiments (Figure S12). For triphenylamines (***S**-***TPA**, Figure S13a) in MCH (with 15% (v/v) CHCl_3_), similar to the study of ***S-*Zn** under VSC, C–H stretches of ***S**-***TPA** and CHCl_3_ overlap with that of MCH (Figure S13b). We observe that under VSC (Figures S13c,d), the *T*_e_ was lowered by ∼5 °C (Figure S14). The *T*_e_ values of different supramolecular systems measured in cuvette (*T*_e, Cuvette_)), NON- (*T*_e, NON-cavity_), OFF-resonance (or Detuned; *T*_e, OFF-res/Detuned_), and ON-resonance (*T*_e, ON-res_) cavities are tabulated in Table 1, and the results confirm the effects of VSC on supramolecular polymerization. Note that the concentration of stock solution and difference in the batches of supramolecular monomer and solvents can lead to some variability in measurements.

**Table 1:** Comparison of *T*_e_ for the Supramolecular Polymerization of *S*-Zn, *S*-TPA, and *S*-T_N_ under Different Conditions

**Conc** (μM)	***T*_e, Cuvette_** (°C)	***T*_e, Noncavity_** (°C)	***T*_e, OFF-res/Detuned_** (°C)	***T*_e, ON-res_** (°C)	**Δ*T***(°C)
***S-*Zn in MCH**					
50	70 ± 1	67 ± 2	67 ± 2	67 ± 2	No change
***S-*Zn in MCH-*d*_14_**					
50	69 ± 1	67 ± 2	67 ± 2	67 ± 2	No change
***S-*Zn in MCH with 1% (v/v) CHCl**_**3**_					
20	66 ± 1	62 ± 1	62 ± 1	57 ± 1	∼5
50	69 ± 1	67 ± 2	67 ± 2	57 ± 2	∼10
80	73 ± 1	72 ± 2	67^a^ ± 2	62 ± 2	∼10
***S-*Zn in MCH-*d*_14_with 1% (v/v) CDCl**_**3**_					
50	67 ± 1	67 ± 2	67 ± 2	67 ± 2	No change
***S-*T_N_in TeCE**					
2200	67 ± 1	67 ± 2	67 ± 2	67 ± 2	No change
***S-*TPA in MCH with 15% (v/v) CHCl**_**3**_					
400	64 ± 1	62 ± 2	62 ± 2	57 ± 2	∼5

a Detuned cavity.

### Switching between ON- and OFF-Resonance Conditions

These intriguing results on the impact of the VSC on ***S*****-Zn** polymerization prompted us to explore the system under kinetic control by tuning the cavity thickness to reach successive ON- and OFF-resonance conditions. Since monomeric and aggregate states are favored at higher temperatures under ON- and OFF-resonance conditions, respectively, we chose 60 °C as the temperature to achieve this.

**Figure 5:**
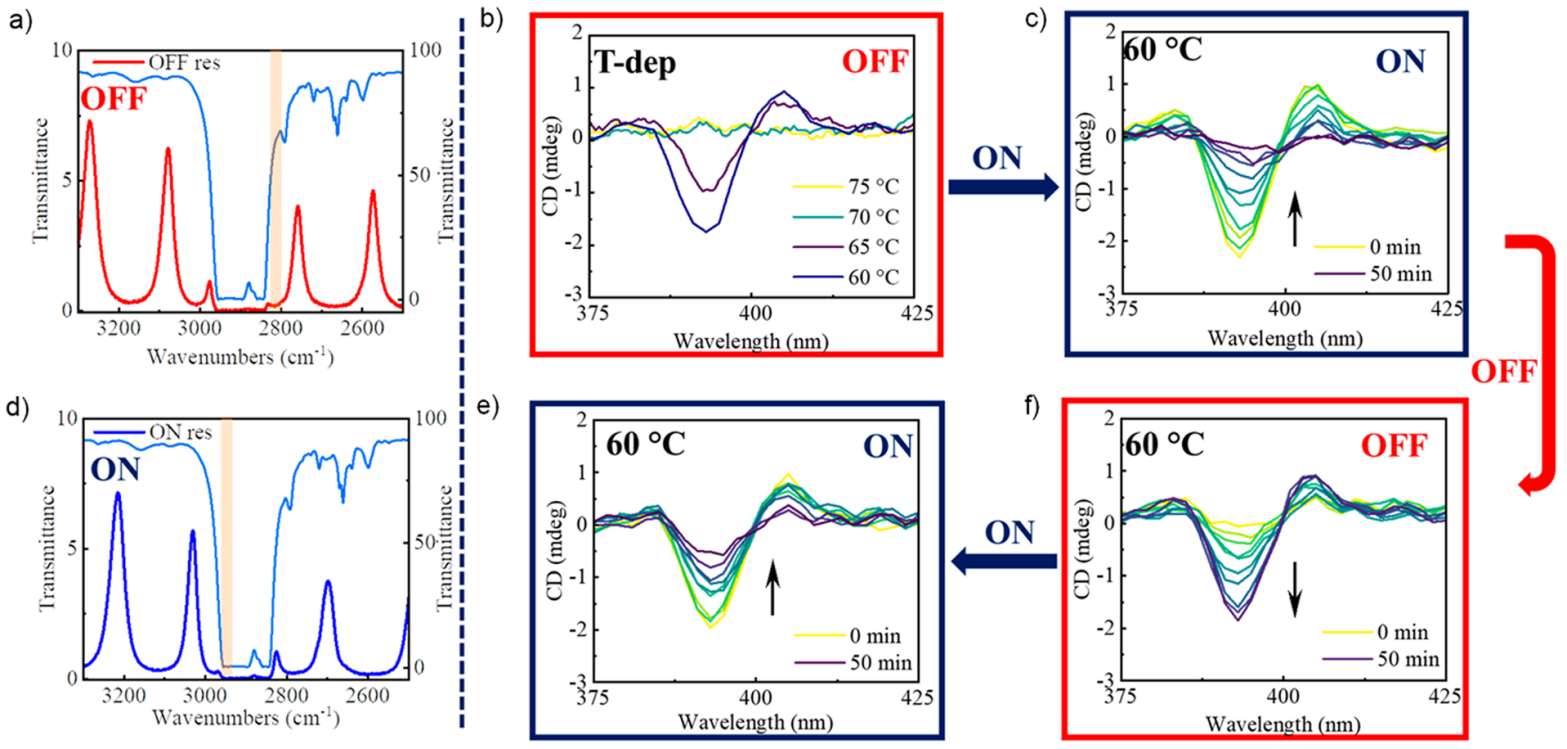
FT-IR transmission spectra of (a) OFF- and (d) ON-resonance cavities. Orange line overlaps with the calculated optical mode, which has been coupled to the vibrational band of MCH, and the light-blue spectrum corresponds to the FT-IR spectrum of MCH. (b) T-dependent CD spectra (from 75 to 60 °C) of *S***-Zn** in MCH with 1% (v/v) CHCl_3_, recorded in an OFF-resonance cavity. At 60 °C, the optical mode is tuned to be in resonance, (c) bisignate CD signal starts to disappear. (f) The cavity is further tuned to the OFF-resonance, and the CD signal starts to reappear. (e) Next, the cavity is tuned back to ON-resonance condition, and the CD signal disappears (for c–f, CD spectra are measured every 5 min for 50 min).

For this purpose, first in an OFF-resonance cavity (Figures 5a), a 50 μM solution of ***S-*Zn** in MCH with 1% (v/v) CHCl_3_ was cooled from 75 to 60 °C (Figure 5b). Since *T*_e_ in the OFF-resonance condition is 65 °C, H-aggregates are formed, which is clear from the appearance of bisignate CD signal at 393 nm (Figure 5b). Soon after, the optical modes are tuned by varying the cavity path length, so that they are in resonance with the vibrational band of MCH at 2900 cm^–1^ (Figure 5d). The bisignate CD signal at 393 nm starts to disappear, thus revealing the destabilization of H-aggregates under the VSC (Figure 5c). After following the evolution of CD spectra in the ON-resonance cavity for 50 min (CD spectra measured every 5 min), the cavity is tuned back to the OFF-resonance condition and is followed for 50 min. We observed that the CD signal at 393 nm starts to appear again (Figure 5f). Next, the optical cavity is tuned back to the strongly coupled condition, and we see the disappearance of the CD signal again (Figure 5e).

**Figure 6:**
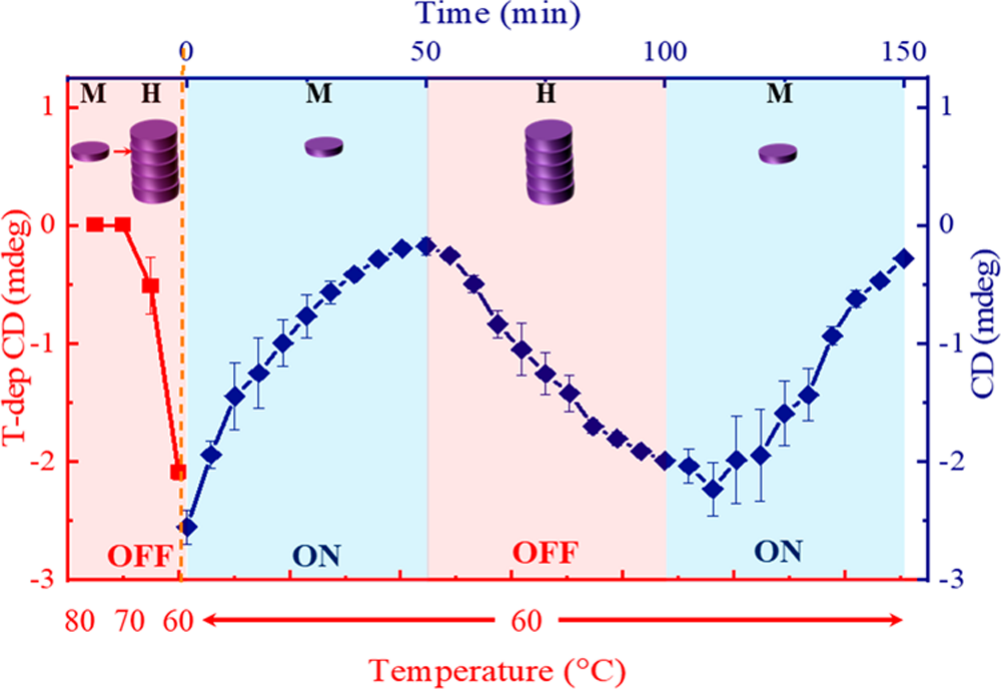
CD signal at 393 nm (blue trace, right axis) corresponding to the H-aggregates of ***S-*Zn** formed in MCH with 1% (v/v) CHCl_3_, measured in a cavity tuned in and out of resonance, is plotted as a function of time. The left axis shows the cooling curve (red trace) followed in the OFF-resonance cavity.

In Figure 6, the CD at 393 nm is plotted as a function of time. The CD intensity every 5 min is plotted as the average of the plots shown in Figure S15. It is clear that VSC can be used to switch between the states of supramolecular polymerization formed under ON- and OFF-resonance conditions by controlling vacuum fluctuations. This unique approach can be further extended to complicated chemical systems, where we can switch between different states merely by tuning the optical mode in and out of resonance with the molecular transitions.

## Outlook and Conclusion

We have demonstrated that the temperature of elongation (*T*_e_) of supramolecular polymerizations can be lowered by ∼10 °C by coupling the C–H stretching frequencies of the solute, bad, and good solvent. Strong coupling of the solvent mixture and monomers leads to the modification of the solute–solute, solute–solvent, and solvent–solvent interactions. As a matter of fact, strong coupling enhances dispersion interactions due to the coherent and collective coupling of molecules to the optical mode.^17,22,34,35^ We envision that a thorough understanding of the effect of the VSC will also enable the control of pathway complexity in supramolecular polymerization. Furthermore, the switching between monomeric and H-aggregate states indicates that different states of supramolecular polymerization can be accessed by controlling the vacuum fluctuations simply by tuning the optical cavity in and out of resonance. These experiments can be further adapted to life-inspired out-of-equilibrium supramolecular systems driven by chemical or real photons as input.^36^ However, the supramolecular oscillations reported so far are damped.^37^ In the future, VSC experiments can also be tailored to study dissipative supramolecular polymerization to generate sustained oscillations via the VSC.

## Supporting Information Available

Materials and methods, and additional control experiments. (PDF)
