# Supplementary material for: Consequences of Vibrational Strong Coupling on Supramolecular Polymerization of Porphyrins
Source: J Am Chem Soc. 2024 Apr 20;146(17):12130–7. doi: 10.1021/jacs.4c02267 (PMC11066862; doi:10.1021/jacs.4c02267)
Supplement: Supplementary file 1 — ja4c02267_si_001.pdf [file ja4c02267_si_001.pdf]

Supporting Information for

Consequences of Vibrational Strong Coupling on Supramolecular Polymerization of Porphyrins

Kripa Joseph[1], Bas de Waal[1], Stef A. H. Jansen[1], Joost J. B. van der Tol[1], Ghislaine Vantomme[1] and E. W. Meijer[1]*

[1]Institute for Complex Molecular Systems, Laboratory of Macromolecular and Organic Chemistry, Eindhoven University of Technology, PO Box 513, 5600 MB, Eindhoven, The Netherlands.

TABLE OF CONTENTS

**1.**	**Materials and methods**	**S02**
	**1.1. Materials**	**S02**
	**1.2. Spectroscopy**	**S02**
	**1.3. Sample preparation**	**S02**
**2.**	**Supramolecular polymerization of *S*-Zn in 100% MCH**	**S03**
**3.**	**Fabrication of optical cavity**	**S03**
**4.**	**Additional control experiments for the effect of VSC.**	
	**4.1. Negligible effect of PVA on supramolecular polymerization of *S*-Zn.**	**S05**
	**4.2. Effect of VSC on supramolecular polymerization of *S*-Zn in 100% MCH**	**S06**
	**4.3. FT-IR transmission spectrum of non-deuterated ($CHCl_3$) and deuterated ($CDCl_3$) chloroform.**	**S06**
	**4.4. VSC dependency on solute concentrations (*S*-Zn in MCH with 1% (v/v) $CHCl_3$).**	**S07**
	**4.5. Supramolecular polymerization of *S*-Zn in deuterated solvents under VSC.**	
	**4.5.1. Effect of VSC on *S*-Zn in MCH-d_{14} (without $CDCl_3$)**	**S08**
	**4.5.2. Effect of VSC on *S*-Zn in MCH-d_{14} (with 1% (v/v) $CDCl_3$)**	**S10**
	**4.6. Supramolecular polymerization of triazines in optical cavities**	**S11**
	**4.7. Supramolecular polymerization of triphenylamines in optical cavities.**	**S13**
**5.**	**ON-OFF cycles of the supramolecular polymerization of *S*-Zn.**	**S15**

1. Materials and methods

1.1. Materials

Methylcyclohexane (MCH, >99%) was purchased from TCI Europe, chloroform ($CHCl_3$) from Biosolve, and 1,1,2,2-tetrachloroethane (TeCE, 98.5%) from Thermo Fischer Scientific. Other solvents including the methylcyclohexane-d_{14} (99.5%) and chloroform-d (99.8%) were purchased from Sigma-Aldrich. All the solvents were used without further purification.

The synthesis of the monomers - chiral zinc-porphyrins (***S*-Zn**), triphenylamines (***S*-TPA**), and triazines (***S*-T_N**), were reported in the previous publications. [1–3]

1.2. Spectroscopy

Supramolecular polymerization of ***S*-Zn** was monitored by recording electronic circular dichroism (ECD) and absorption spectra with a JASCO J-815 spectropolarimeter in cuvette, and NON-, OFF- and ON- resonance cavities. The measurement parameters for sensitivity, scanning rates, slit width and ranges were chosen appropriately for the cavity experiments. The ECD signal at 393 nm was followed to extract the elongation temperature of ***S*-Zn**. The spectropolarimeter was equipped with a SPECAC temperature controller and the ramp rate was fixed at 100 °C hr^{-1} (~1.7 °C min^{-1}) for following supramolecular polymerization.

Fourier-Transform Infrared (FT-IR) transmission spectra of NON- and Fabry-Perot cavities were recorded with a Shimadzu IR-Tracer 100 Fourier-Transform Infrared spectrometer coupled to a TGS detector. The spectra were acquired with 2 cm^{-1} resolution and averaged over 32 scans for the samples. For temperature-dependent measurements, tuning of cavity was ensured by recording FT-IR transmission spectra at every temperature after measuring ECD spectra. If there is any shift due to thermal effect, the cavity is tuned back to the appropriate resonance condition and the same protocol is continued throughout the temperature range to follow the supramolecular polymerization. Aforementioned method was adapted for the supramolecular polymerization studies of ***S*-TPA**, and ***S*-T_N** under VSC.

1.3. Sample preparation

Stock solutions (200 μM) of ***S*-Zn** in $CHCl_3$ were prepared by weighing the compound into a vial and adding the appropriate amount of solvent. The required volume of the stock solution was taken, and the solvent was evaporated. $CHCl_3$ being a polar (or good) solvent breaks the aggregates. The pre-prepared solvent composition (MCH-$CHCl_3$) was then added, and the solution was sonicated until complete dissolution. The ***S*-Zn** solution was further equilibrated in a cuvette, and injected into the NON-, OFF-resonance and ON-resonance cavities maintained at a temperature of 75°C. Similar protocol was used for the measurements of ***S*-TPA**, and ***S*-T_N**.

2. Supramolecular polymerization of *S*-Zn in 100% MCH

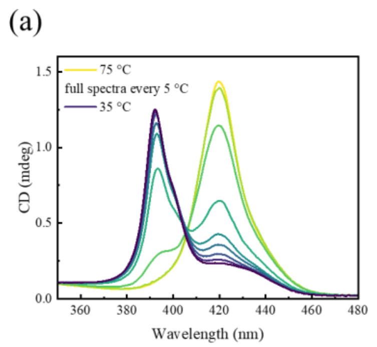

Figure S1: Cuvette measurements: (a) VT-absorption, (b) VT-ECD spectra and (c) cooling curve of ***S*-Zn** (conc = 50 μM) in MCH.

3. Fabrication of optical cavity

IR transparent windows (BaF_2) windows, tunable microfluidic cells and 12 μm Mylar spacers were purchased from Specac. The windows were sputtered with 10 nm of Au in Quorum Q150T Plus turbomolecular coater and then a 100 nm thick insulating layer was spin-coated with a solution of PVA in water 4% (w/w) at 3000 rpm in a spin-coater. The Au mirrors were separated by a 12 μm Mylar spacer and were assembled into the microfluidic cell which is also compatible for temperature-controlled measurements. The cavities were tuned to different vibrational modes of solvents by tightening or loosening the screws. For NON-cavity experiments, the substrates were prepared by directly spin-coating PVA on BaF_2 windows. In OFF-resonance cavities, the optical modes are tuned away from the vibrational bands, acting as an additional reference. Both NON- and OFF-resonance cavities take into account the artifacts contributed by the insulation film, physical confinement, or Au mirrors, into consideration.

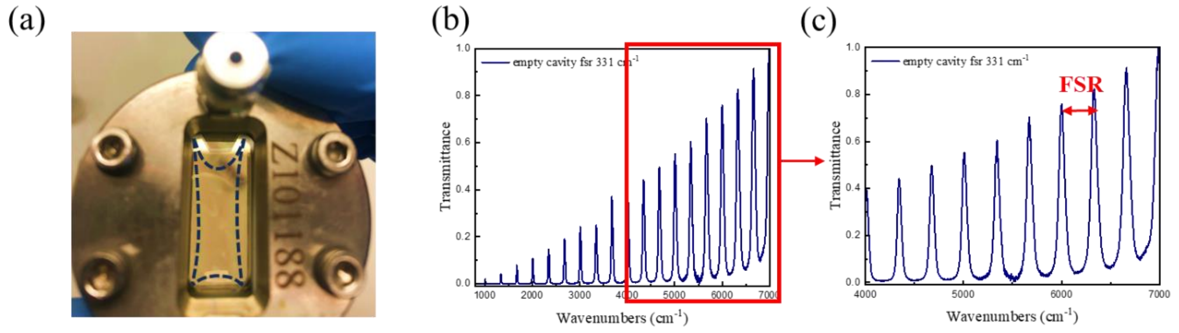

Figure S2: (a) Image of a tunable microfluidic cell with a tuned optical cavity. Blue dashed line is a guide to the eye. FT-IR transmission spectra of the (b) empty cavity which is zoomed in (c) with FSR = 331 cm^{-1}.

The optical cavities are tuned such that the sweet spot (Figure S2a, reflecting the region of resonance and parallelism of the mirrors) is approximately 85-90% of the total area, so that the source beam size of the

spectrometer is smaller than the tuned area of the cavity. This is to avoid the contribution of the supramolecular system formed in the detuned region of the cavity to the spectroscopic measurements.

$$FSR = \frac{10^4}{2.n.l} \qquad \text{------------------------}(I)$$

Based on the refractive index (n) of the solvent and spacing between the mirrors (l), the FSR (or free spectral range, equation I) can be calculated a priori, and the empty cavity (Figure S2b,c) is thus pre-tuned for the cavity measurements.

4. Additional control experiments

4.1. Negligible effect of PVA on supramolecular polymerization of S-Zn.

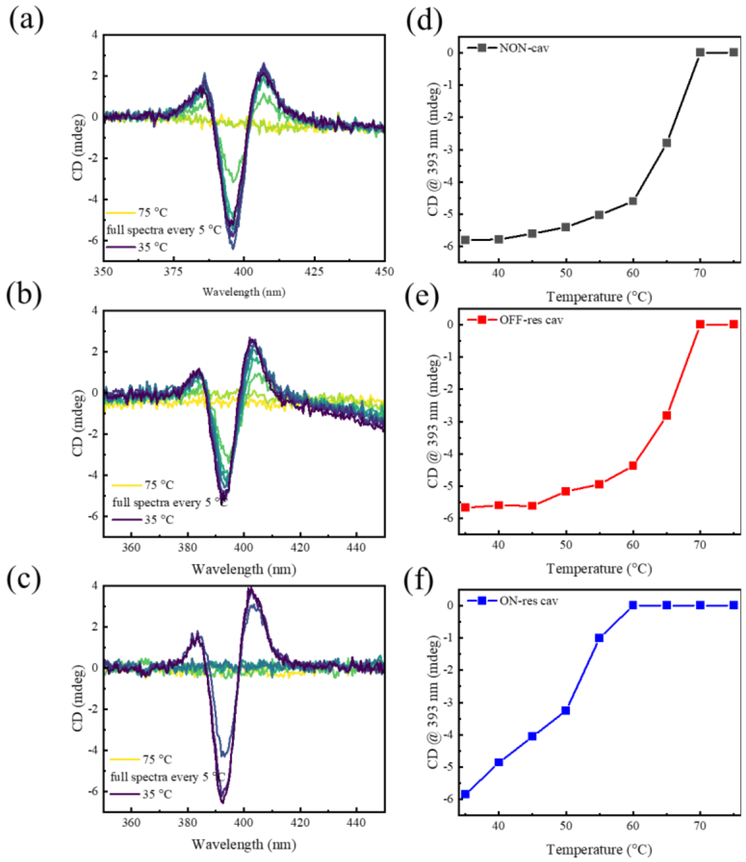

Figure S3: VT-ECD spectra of ***S*-Zn** (conc = 50 μM) measured in NON-, OFF-resonance and ON-resonance cavities coated with SiOx, are shown in (a), (b) and (c) and the corresponding cooling curves plotted as a function of concentration are shown in (d), (e) and (f). All the spectra are measured in the interval of 5 °C from 75 to 35 °C with a cooling rate of 1.7 °C min^{-1}.

To check whether PVA acts as a hydrogen-bond scavenger, we repeated the experiments of ***S*-Zn** in MCH (with 1% (v/v) of $CHCl_3$) in NON-, OFF-resonance, and ON-resonance cavities evaporated with SiOx as the insulation layer. We observed that T_e is still lowered by ~10 °C, thus nullifying the effect of PVA on the supramolecular polymerization of ***S*-Zn**.

4.2. Effect of VSC on supramolecular polymerization of S-Zn in 100% MCH

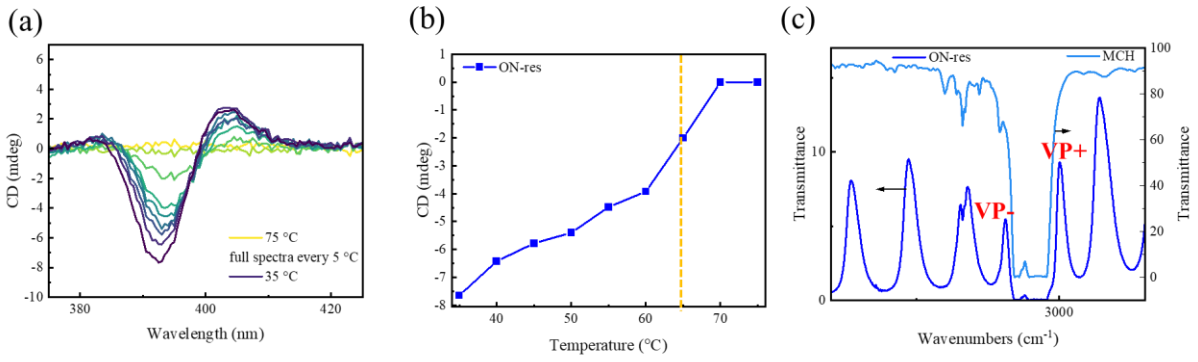

Figure S4: (a) VT-ECD spectra and (b) the corresponding cooling curve of ***S*-Zn** (conc = 50 μM) in MCH measured in ON-resonance cavity. (c) FT-IR transmission spectra of ON-resonance cavity showing the vibro-polaritonic states formed.

4.3.FTIR transmission spectra of non-deuterated ($CHCl_3$) and deuterated ($CDCl_3$) chloroform

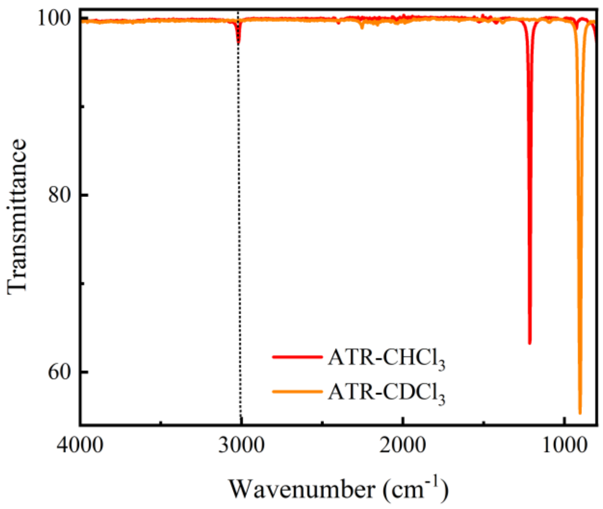

Figure S5: FT-IR transmission spectra of $CHCl_3$ (red trace) and $CDCl_3$ (orange trace). The black dotted line shows the C-H stretch of $CHCl_3$, which can also be coupled to the optical mode via cooperative effect due to the overlap with the C-H stretches of MCH.

4.4. VSC dependency on solute concentration (*S*-Zn in MCH with 1% (v/v) $CHCl_3$)

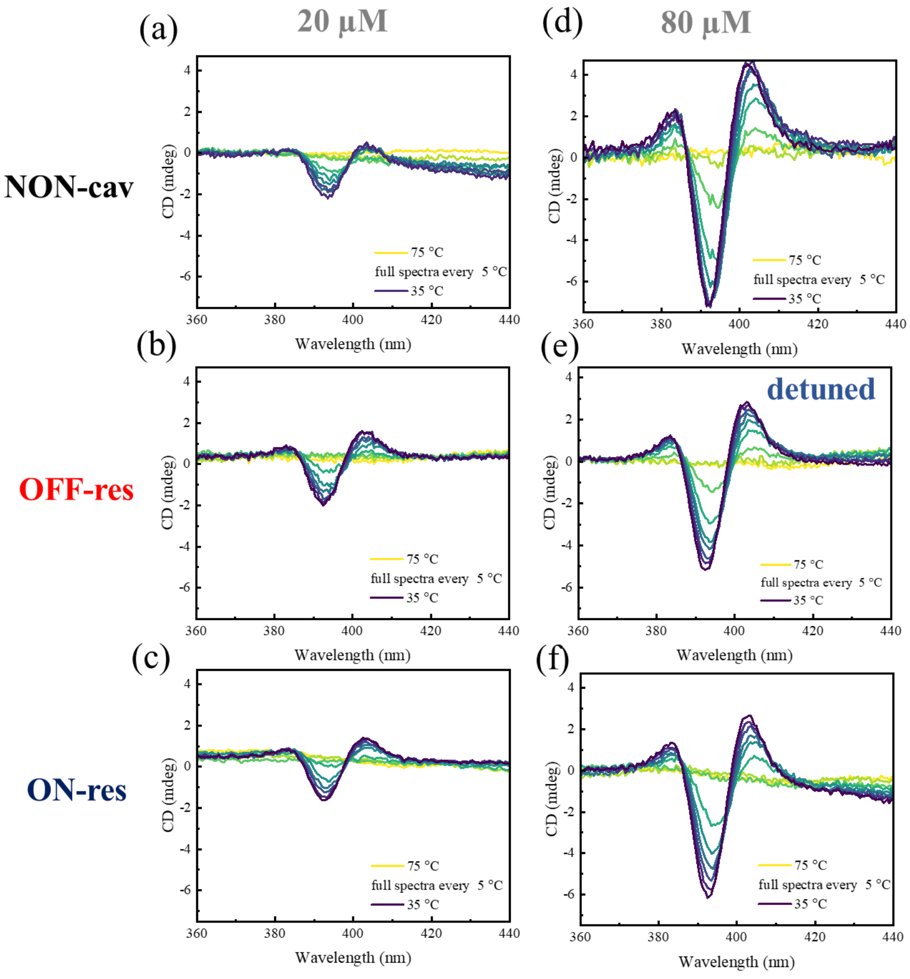

Figure S6: VT-ECD spectra of ***S*-Zn** (conc = 20 μM) in MCH with 1% (v/v) $CHCl_3$, measured in (a) NON-, (b) OFF-resonance, and (c) ON-resonance cavities. VT-ECD spectra of ***S*-Zn** (conc = 80 μM) in MCH with 1% (v/v) $CHCl_3$, measured in (d) NON-, (e) OFF-resonance, and (f) ON-resonance cavities.

4.5. Supramolecular polymerization of *S*-Zn in deuterated solvents under VSC.

4.5.1. Effect of VSC on *S*-Zn in MCH-d14 (without $CDCl_3$)

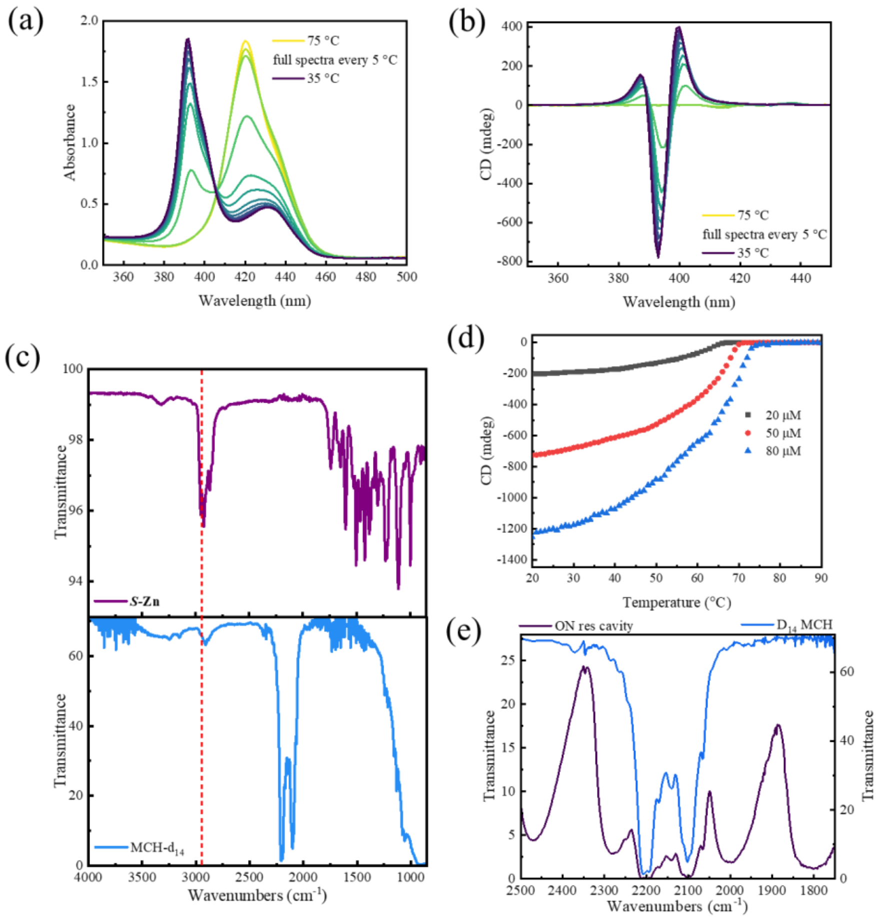

Figure S7: (a) VT-absorption and (b) VT-ECD spectra of ***S*-Zn** in MCH-d14 measured in the interval of 5 °C from 75 to 35 °C with a cooling rate of 1 °C min^{-1} in cuvette. (c) FT-IR transmission spectra of ***S*-Zn** (top) and MCH (bottom), red dashed lines shows that the vibrational bands of solute and solvent (around 2900 cm^{-1}) are not overlapping. Therefore, cooperative coupling is not possible. (d) ECD cooling curves of ***S*-Zn** plotted as a function of concentration. (e) FT-IR transmission spectra of ON resonance cavity wherein C-D vibrational bands of MCH-d14 are coupled to the optical mode.

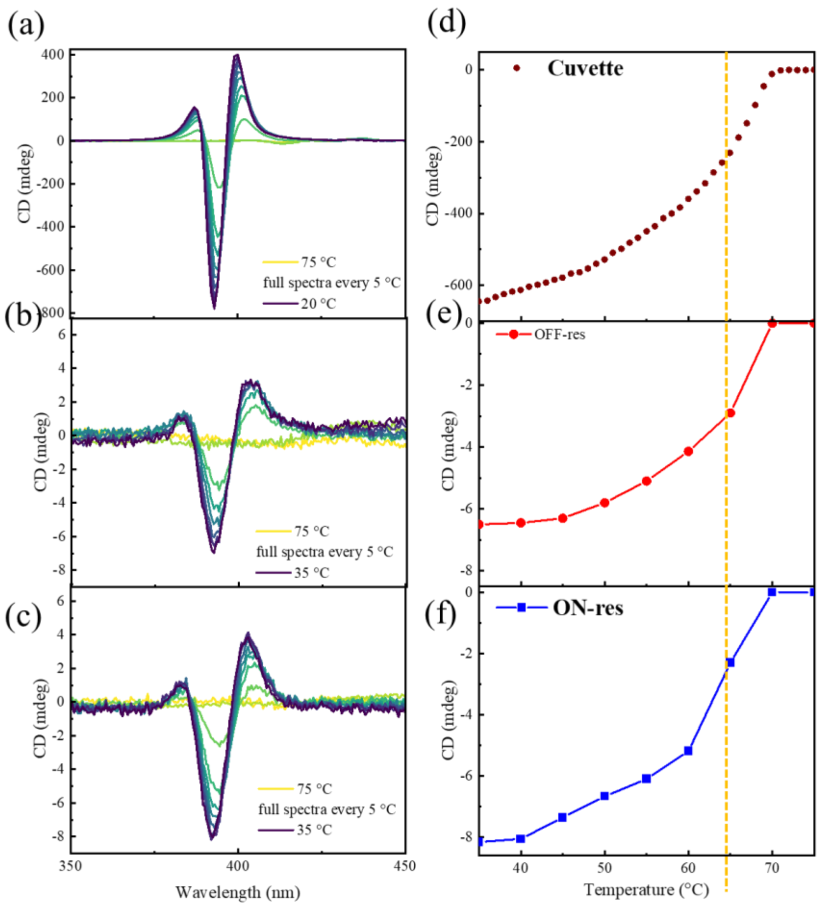

Figure S8: VT-ECD spectra of ***S*-Zn** (conc = 50 μM) in MCH-d_{14} measured in (a) cuvette, (b) OFF- and (c) ON-resonance cavities. The corresponding cooling curves are plotted in (d), (e) and (f). The spectra in ON- and OFF resonance cavities are measured in the interval of 5 °C from 75 to 35 °C with a cooling rate of 1.7 °C min^{-1}. Yellow dashed line goes through the T_e observed in OFF- and ON-resonance cavities.

4.5.2. Effect of VSC on ***S*-Zn** in MCH-d_{14} (with 1% (v/v) $CDCl_3$)

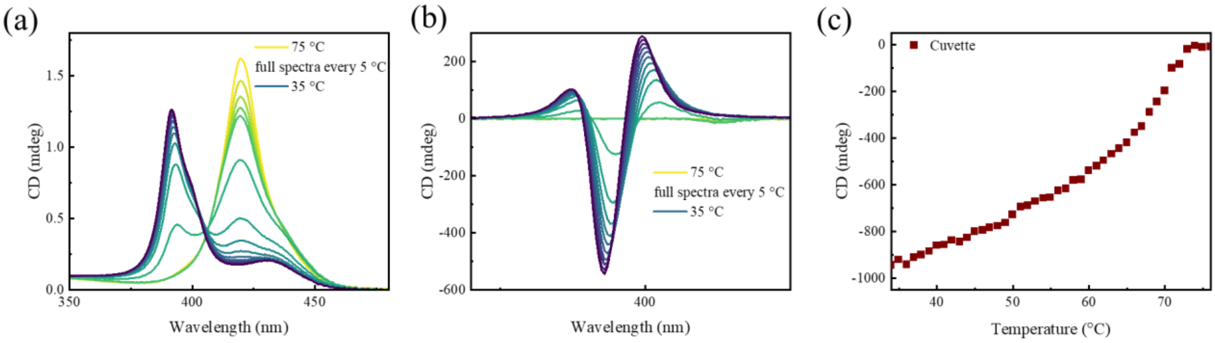

Figure S9: (a) VT-absorption (b) VT-ECD spectra and (c) cooling curve of ***S*-Zn** (conc = 50 μM) in MCH-d_{14} with (1% (v/v) $CDCl_3$) measured in cuvette.

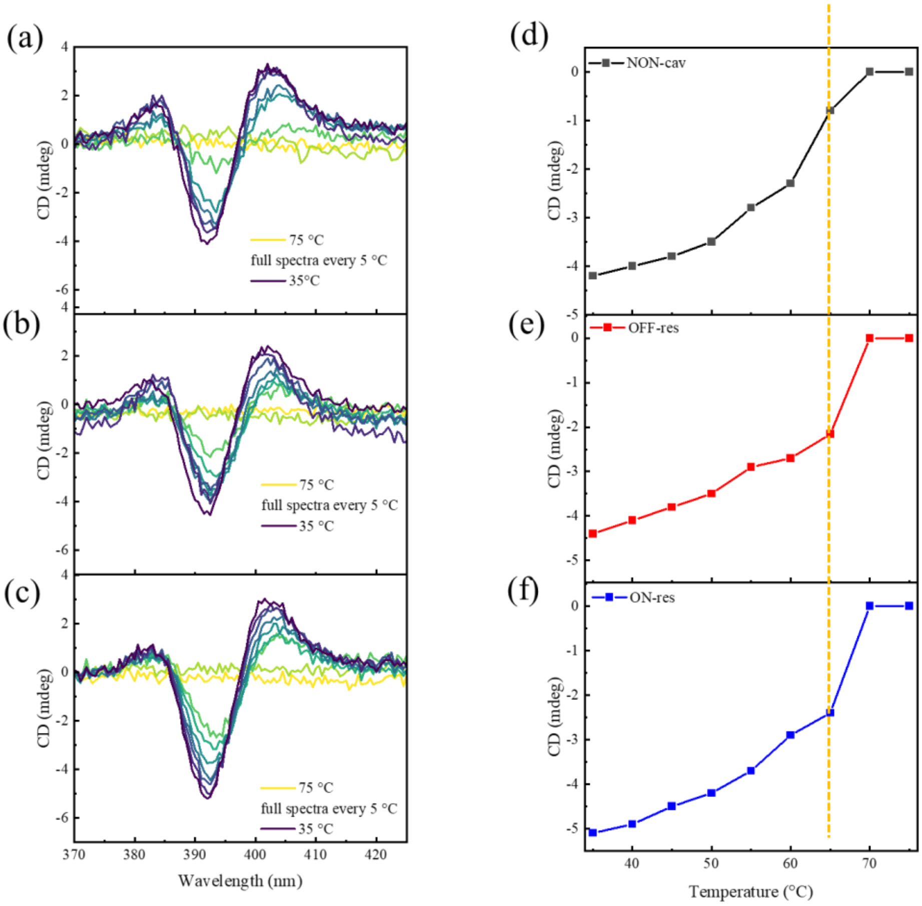

Figure S10: VT-ECD spectra of ***S*-Zn** (conc = 50 μM) in MCH-d_{14} with (1% (v/v) $CDCl_3$) measured in (a) cuvette, (b) OFF-resonance, and (c) ON-resonance cavities. The corresponding cooling curves are plotted in (d), (e) and (f). The spectra in ON- and OFF resonance cavities are measured in the interval of 5 °C from 75 to 35 °C with a cooling rate of 1.7 °C min^{-1}. Yellow dashed line goes through the T_e observed in OFF- and ON-resonance cavities.

4.6. Supramolecular polymerization of triazines in optical cavities

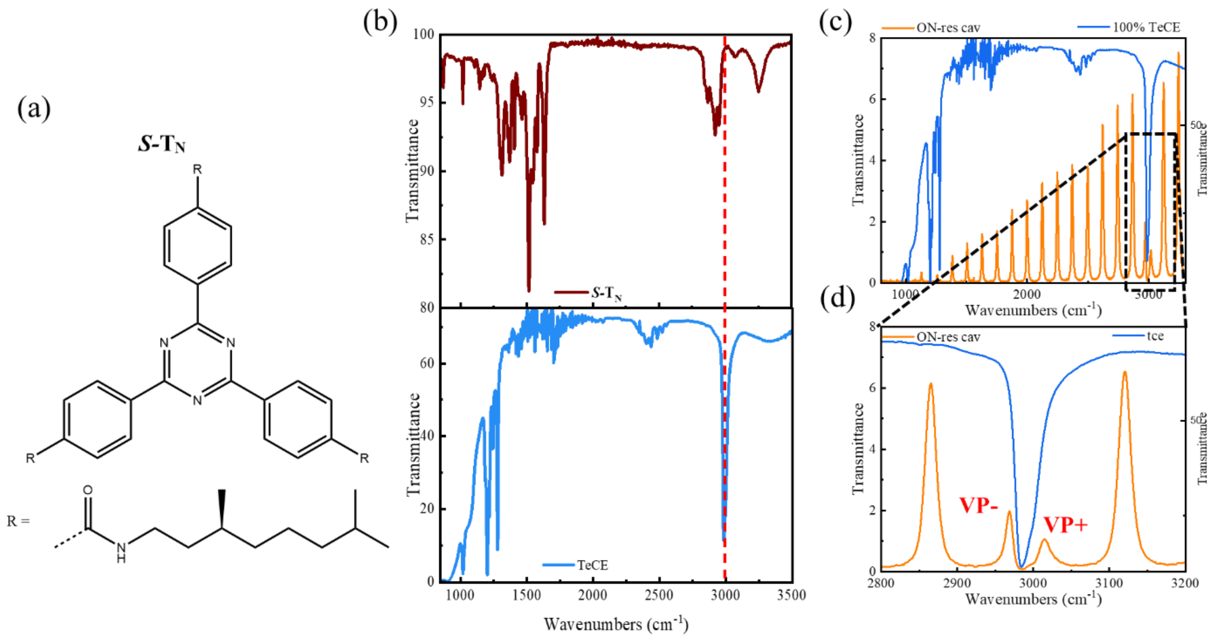

Figure S11: (a) Molecular structure of supramolecular monomer ***S*-T**$_N$. (b) FT-IR transmission spectra of ***S*-T**$_N$ (top) and TeCE (bottom), red dashed line shows that the vibrational bands of solute and solvent (around 3000 cm^{-1}) are not overlapping. Therefore, cooperative coupling is not possible. (c) FT-IR transmission spectra of ON-resonance cavity, vibro-polaritonic states are zoomed in (d).

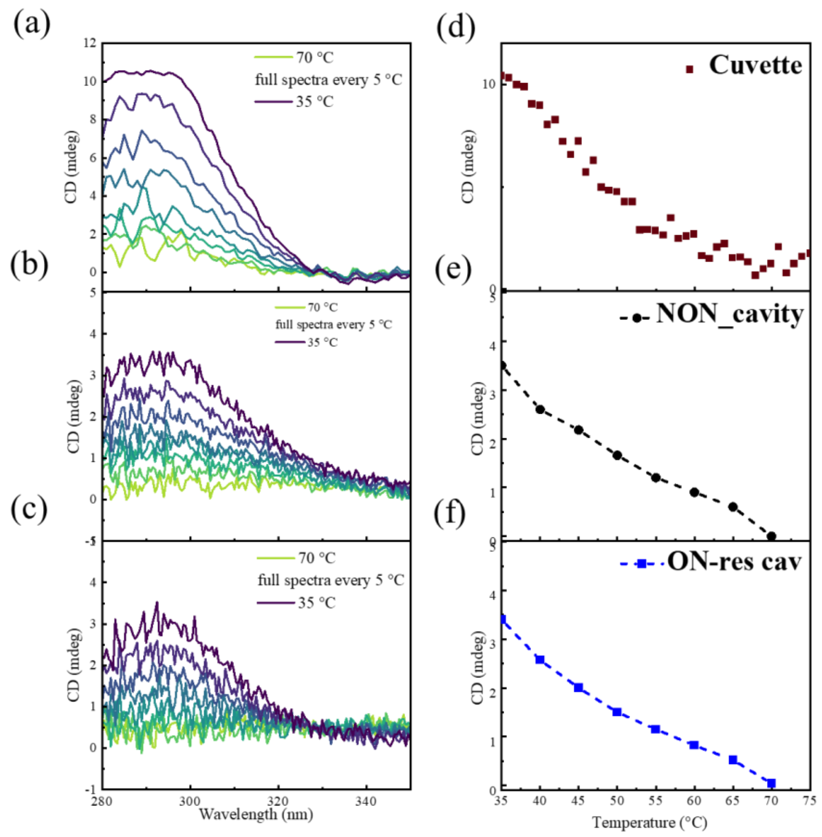

Figure S12: VT-ECD spectra of ***S*-T$_N$** (conc = 2200 μM) measured in cuvette, OFF-resonance and ON-resonance cavities are shown in (a), (b) and (c) and the corresponding cooling curves are shown in (d), (e) and (f). All the spectra are measured in the interval of 5 °C from 70 to 35 °C with a cooling rate of 1.7 °C min^{-1}. Yellow dashed line goes through the T_e observed under strong coupling condition.

4.7. Supramolecular polymerization of triphenylamines in optical cavities

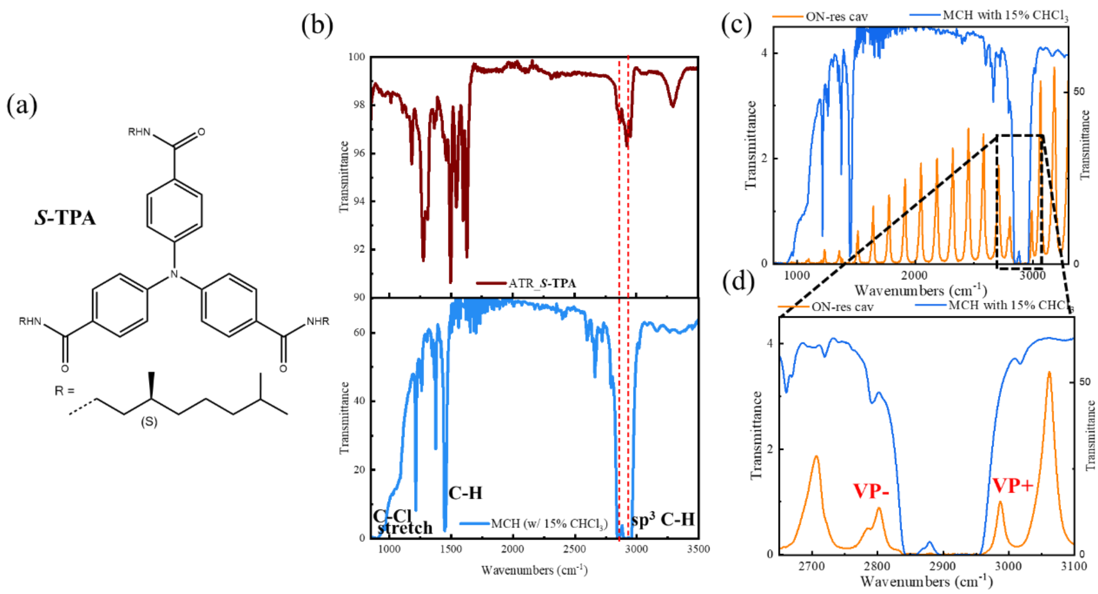

Figure S13: (a) Molecular structure of supramolecular monomer ***S*-TPA**. (b) FT-IR transmission spectra of ***S*-TPA** (top) and MCH (bottom, with 15% (v/v) $CHCl_3$), red dashed lines show the overlapping bands between the solute and solvent. (c) FT-IR transmission spectra of ON-resonance cavity, vibro-polaritonic states are zoomed in (d).

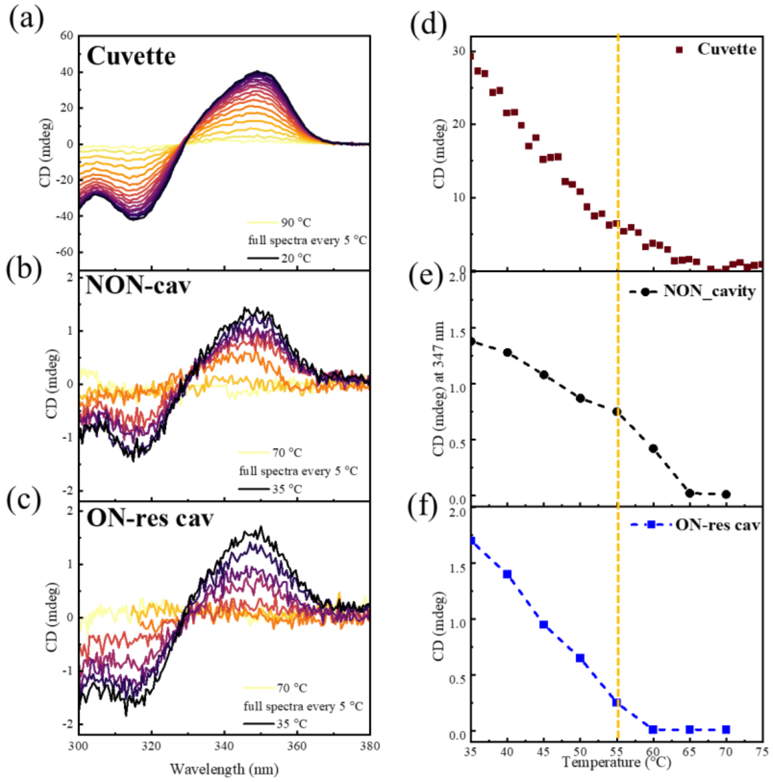

Figure S14: VT-ECD spectra of ***S*-TPA** (conc = 400 μM) measured in cuvette, OFF-resonance and ON-resonance cavities are shown in (a), (b) and (c) and the corresponding cooling curves are shown in (d), (e) and (f). All the spectra are measured in the interval of 5 °C from 70 to 35 °C with a cooling rate of 1.7 °C min^{-1}. The yellow dashed line goes through the T_e observed under strong coupling condition.

5. **ON-OFF cycles of the supramolecular polymerization of *S*-Zn.**

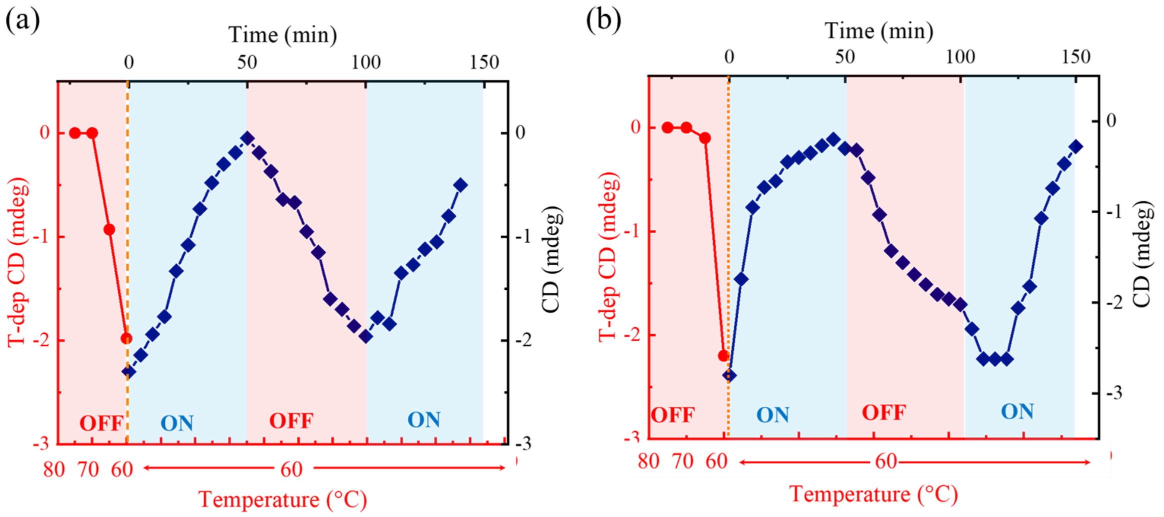

Figure S15: Two different sets (recorded on different days with freshly prepared solutions) of ON-OFF cycles and average (of CD intensity every 5 minutes) of both plots is shown in Figure 6.

References

[1] H. Su, S. A. H. Jansen, T. Schnitzer, E. Weyandt, A. T. Rösch, J. Liu, G. Vantomme, E. W. Meijer, *J. Am. Chem. Soc.* **2021**, *143*, 17128–17135.

[2] B. Adelizzi, I. A. W. Filot, A. R. A. Palmans, E. W. Meijer, *Chem. Eur. J.* **2017**, *23*, 6103–6110.

[3] F. Helmich, C. C. Lee, M. M. L. Nieuwenhuizen, J. C. Gielen, P. C. M. Christianen, A. Larsen, G. Fytas, P. E. L. G. Leclère, A. P. H. J. Schenning, E. W. Meijer, *Angew. Chem. Int. Ed.* **2010**, *49*, 3939–3942.
